# Hemophagocytic Lymphohistiocytosis in the Setting of Therapy-Induced Acute Myeloid Leukemia: An Autopsy Report

**DOI:** 10.3390/diseases10030054

**Published:** 2022-08-19

**Authors:** Hisham F. Bahmad, Samantha Gogola, Mohamad K. Elajami, Carole Brathwaite, Amilcar A. Castellano-Sánchez, Vathany Sriganeshan, Yumna Omarzai

**Affiliations:** 1 Department of Pathology and Laboratory Medicine, Mount Sinai Medical Center, Miami Beach, FL 33140, USA; 2 Herbert Wertheim College of Medicine, Florida International University, Miami Beach, FL 33199, USA; 3 Department of Internal Medicine, Mount Sinai Medical Center, Miami Beach, FL 33140, USA; 4 Department of Pathology, Nicklaus Children’s Hospital, Miami Beach, FL 33155, USA

**Keywords:** therapy-induced acute myeloid leukemia, hemophagocytic lymphohistiocytosis, hemorrhagic pericardial effusion

## Abstract

Hemophagocytic lymphohistiocytosis (HLH) is a life-threatening hyper-inflammatory disorder that occurs due to immunologic dysregulation. HLH can be primary (hereditary) or secondary to infections, autoimmune diseases, immune deficiencies, metabolic diseases, drugs, or malignancies. Lymphoid neoplasms mostly accompany malignancy-associated HLH. We present a case of a 12-year-old boy with a history of precursor B lymphoblastic leukemia (B-ALL), who subsequently developed chemotherapy-induced acute myeloid leukemia (t-AML). The patient was admitted for febrile neutropenia and initial laboratory tests revealed hemophagocytic lymphohistiocytosis (HLH). The hospital course was complicated by multiple infections and septic shock. The patient received several broad-spectrum antimicrobials, dexamethasone, as well as a pericardial drain to drain the hemorrhagic pericardial effusion. Despite intervention, the patient expired, and an autopsy was performed. We provide a synopsis of the main autopsy findings.

## 1. Introduction

Hemophagocytic lymphohistiocytosis (HLH) is a life-threatening hyper-inflammatory disorder that is due to immunologic dysregulation. The diagnosis of HLH is based on standard criteria proposed by Henter et al. in 2004 (Table 1) [1]. According to the standard diagnostic criteria proposed in 2004 by Henter et al., diagnosis can be made either based on molecular studies or based on clinicopathologic criteria where five out of eight points need to be met. HLH can be divided into two groups: primary (hereditary) and secondary [2,3]. Primary HLH is often observed in childhood in association with a multitude of genetically heterogeneous disorders and it is typically triggered by common infections [4]. The secondary form can occur at any age and typically occurs subsequent to immunosuppression, such as that seen in malignancy [5]. Malignancy-associated HLH is further subdivided into the malignancy-triggered form and chemotherapy-associated HLH [6]. In chemotherapy-associated HLH, direct cytotoxic effects, secondary infection, or the hyper-inflammatory state of the treatment serve as the initiating triggers [3]. More often, both forms of malignancy-associated HLH overlap when therapy is involved, and the exact trigger may not be able to be identified. Malignancy-associated HLH is typically associated with lymphoid neoplasms and leukemias, although its occurrence in association with solid tumors can be seen as well [2,3].

Herein, we present a case of a 12-year-old boy with a history of precursor B lymphoblastic leukemia (B-ALL), who subsequently developed chemotherapy-induced acute myeloid leukemia (t-AML). The patient was admitted for febrile neutropenia. Physical examination and initial laboratory tests yielded a diagnosis of hemophagocytic lymphohistiocytosis (HLH). The hospital course was complicated by multiple infections (herpes simplex virus (HSV)-1 stomatitis, right parotiditis, bilateral myositis, left lower leg fasciitis and phlebitis, and fungal lung infection) and septic shock. The patient also developed hemorrhagic pericardial effusion and expired approximately 1-month post admission. An autopsy was performed at Mount Sinai Medical Center.

## 2. Case Presentation

### 2.1. Clinical History

This is a case of a 12-year-old boy with a history of precursor B lymphoblastic leukemia (ALL) diagnosed at around the age of 5 years, who subsequently developed chemotherapy-induced acute myeloid leukemia (t-AML) with 5q deletion and monosomy 7 at age of 11 years (approximately 11 months prior to the current presentation). The patient had a history of treatment with anthracycline chemotherapy. He was admitted for febrile neutropenia. The patient had no known allergies and procedural history of insertion of tunneled centrally inserted central venous access device with subcutaneous port.

Vital signs on admission showed a temperature of 99.7 °F (37.6 °C), heart rate of 118 bpm, blood pressure (BP) of 89/49 mmHg, respiratory rate of 18 breaths/minute, and SpO2 98%. His body mass index (BMI) was 30.34 kg/m^2^ (height 166.25 cm, weight 83.85 kg). Physical examination on admission showed hepatosplenomegaly. The patient’s blood group was O+. Laboratory results showed pancytopenia with a critically low white blood cell (WBC) count of 600 per µL (reference range 4800–10,800 per µL) and a differential as follows: 3.6% neutrophils (reference range 38.0–74.0%), 73.2% lymphocytes (reference range 27.0–47.0%), 21.4% monocytes (reference range 6.0–10.0%), 0.0% eosinophils (reference range 1.0–4.0%), and 0.0% basophils (reference range 0.0–1.0%). Red blood cell (RBC) count was low at 1.93 million per µL (reference range 4.5–5.3 million per µL), hemoglobin 5.7 g/dL (reference range 13.5–17.5 g/dL), hematocrit 16.2% (reference range 37.0–49.0%), mean corpuscular volume (MCV) 83.9 fL (reference range 78.0–110.0 fL), mean corpuscular hemoglobin (MCH) 29.5 pg (reference range 25.0–31.0 pg), mean corpuscular hemoglobin concentration (MCHC) 35.2 g/dL (reference range 32.0–36.0 g/dL), and red blood cell distribution width (RDW) 15.0% (reference range 11.5–14.5%). The patient also had thrombocytopenia with a platelet count of 110,000 per µL (reference range 150,000–500,000 per µL). Prothrombin time (PT) was high at 28.1 s (reference range 11.6–15.4 s), international normalized ratio (INR) 2.61, and partial thromboplastin time (PTT) > 200 s (reference range 22.8–38.2 s). In addition, ferritin level was raised at 4770 μg/L (reference range 9–125 μg/L). Levels of interleukin (IL)-6 (>400 pg/mL; reference range ≤ 1.8 pg/mL), soluble CD25/sIL-2R (5615 U/mL; reference range 137–838 U/mL), and transaminases were elevated as well. Collectively, the history of fever, splenomegaly, and laboratory findings of pancytopenia, hyperferritinemia (≥500 μg/L), and high soluble CD25 (≥2400 U/mL) yielded the diagnosis of hemophagocytic lymphohistiocytosis (HLH) with at least five of the eight diagnostic criteria of HLH being met [1] (Table 1).

The hospital course was complicated by herpes simplex virus (HSV)-1 stomatitis from an ulcer at the tip of the tongue. On day 3 of admission, the patient developed worsening fevers, difficulty breathing, and right cheek swelling and erythema with computed tomography (CT) of the neck showing right parotiditis. Creatine kinase (CK) trended up, peaking at 3340 IU/L (reference range 30.0–150.0 IU/L) on day 7 of admission. Magnetic resonance imaging (MRI) of the lower extremities showed bilateral myositis (mild on right and severe on left), fasciitis of the left lower leg, and possibly left lower extremity phlebitis.

Simultaneously, the patient developed worsening tachycardia, hypotension, and respiratory distress. An echocardiogram showed cardiomegaly and severely depressed biventricular ejection fraction of 33% with worsening pericardial effusion and no significant improvement with vasoactive support or stress hydrocortisone. A pericardial drain was placed yielding hemorrhagic fluid; the patient was intubated and put on venoarterial extracorporeal membrane oxygenation (VA-ECMO) for 3 days.

During hospitalization, the patient had recurring hemorrhagic pericardial effusions. He required multiple blood transfusions, including packed RBCs, fresh frozen plasma (FFP), platelets for hemoglobin < 7 g/dL and platelets < 10,000 per µL, and aggressive diuretics for fluid overload, causing him hypocalcemia and hypokalemia. The patient required frequent electrolyte replacement and a calcium drip. He received one dose of Tocilizumab (humanized monoclonal antibody against IL-6 receptor) on day 15 and continued to receive dexamethasone for HLH management.

On day 35 of admission, procalcitonin peaked at 334.090 ng/mL (reference range 0.000–0.100 ng/mL) and the patient was transferred to the pediatric intensive care unit (PICU) for concerns of septic shock (blood cultures grew *Prevotella melaninogenica* bacteria) in the setting of HLH and airway compromise. He also had a fungal infection of the lungs. For sepsis, he was initially treated with daptomycin, acyclovir, voriconazole, and meropenem, and was given HAT therapy (hydrocortisone, ascorbic acid, and thiamine). Meropenem was continued for *Prevotella* treatment and micafungin was given prophylactically (switched from voriconazole due to AKI).

The patient’s condition was acutely decompensated requiring escalating doses of vasopressors for blood pressure support. He had an acute episode of altered mental status and respiratory failure requiring sedation and paralysis for endotracheal intubation. During this episode, he had an acute change in cardiac rhythm with an electrocardiogram (EKG) showing sinus tachycardia, intraventricular conduction delay, ST-segment, and T-wave abnormalities, compatible with myocardial injury. His troponin trended up to 9.70 ng/mL (reference range 0.00–0.08 ng/mL) and an echocardiogram was obtained showing the increased density of the effusion along the left ventricular (LV) free wall, trivial anterior effusion, and depressed LV systolic function, suggestive of tamponade physiology. The patient expired on day 36 of admission and the autopsy was performed on postmortem day 5.

### 2.2. Autopsy Findings

#### 2.2.1. Cardiovascular System

**Figure 1:**
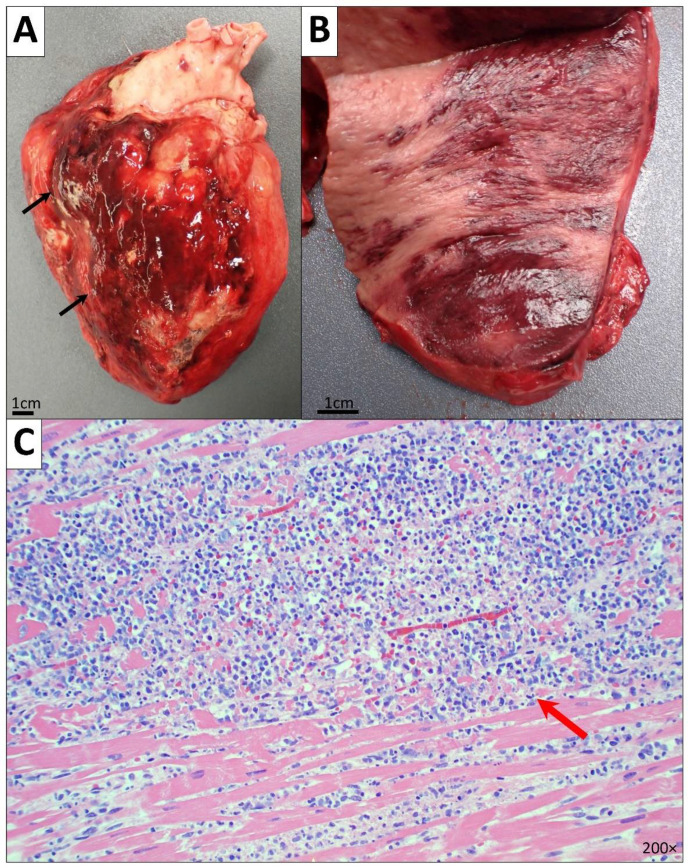
Heart and pericardium. (**A**,**B**) Gross images of the heart and cut surface of the LV showing cardiomegaly and extensive and diffuse hemorrhagic fibrinous and fibrous adhesions (black arrow), hemorrhage and clotted blood, and fibrinous exudates. (**C**) Microscopic image of the LV myocardium showing myocyte hypertrophy, diffuse interstitial fibrosis, AML cell infiltrates (red arrow), and hemophagocytosis (200× magnification).

Examination of the cardiovascular system showed cardiomegaly (451 g; range 128–336 g [7]) with a fatty epicardium. There was a white synthetic pericardiocentesis catheter in place in the pericardial sac. The pericardial sac had extensive and diffuse hemorrhagic fibrinous and fibrous adhesions, hemorrhage and clotted blood, fibrinous exudates (at least 300–400 cc), hemophagocytosis and reactive mesothelial hyperplasia, involving predominantly the anterior wall of the LV. Pericardial fibrous adhesions were also seen between the epicardium and diaphragm (Figure 1). The native coronary arteries, bilateral cardiac chambers, and cardiac valves were grossly and microscopically unremarkable. The aortic arch, ascending aorta, descending aorta, and aortic bifurcation was also unremarkable.

There was biventricular hypertrophy; the LV maximum thickness measured 2.2 cm (normal average: 1.5 cm) and the right ventricle (RV) measured 1.1 cm (normal average: 0.5 cm) in maximum thickness. Microscopically, myocyte hypertrophy and diffuse interstitial fibrosis were seen. Notably, there were acute myeloid leukemic (AML) infiltrates associated with hemorrhage and necrosis extensively involving the pericardial fibroadipose tissue, epicardium, and myocardium including the left ventricle, right ventricle, and interventricular septum (supporting immunohistochemical (IHC) stains were positive for myeloperoxidase (MPO), while negative for CD117). The posteromedial and anterolateral papillary muscles showed sparse AML infiltrates with patchy fibrosis and features consistent with remote ischemic changes.

#### 2.2.2. Respiratory System

Examination of the respiratory system showed bilateral serosanguineous pleural effusion (20 cc on the right and 220 cc on the left). There were fibrinous adhesions between the left lung lobes and the chest wall, pleura, and pericardium. The left lung weighed 480 g and the right lung 730 g (normal range, left: 64–624 g, right: 100–668 g, [7]). Moderate to severe bilateral pulmonary congestion and edema was noted and most pronounced in the right lower lobe, with pleural hemorrhages and petechiae.

**Figure 2:**
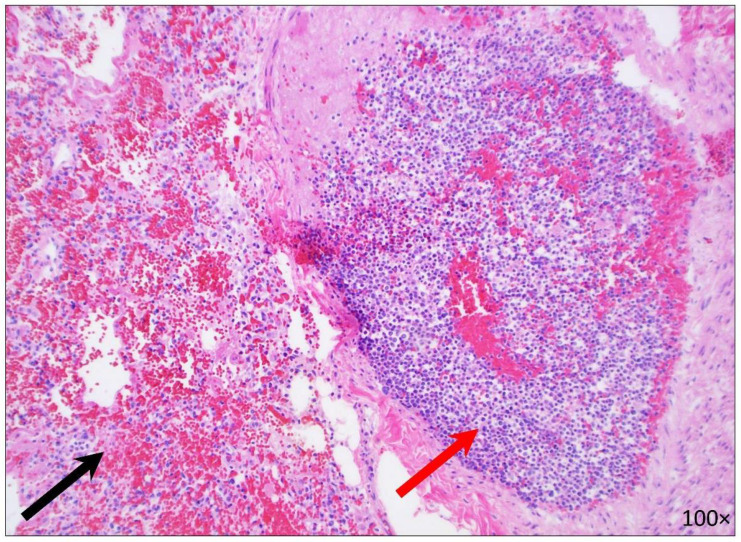
Microscopic image of the lung (RLL) showing intravascular AML infiltrates (red arrow) and diffuse intra-alveolar hemorrhages (black arrow) (100× magnification).

**Figure 3:**
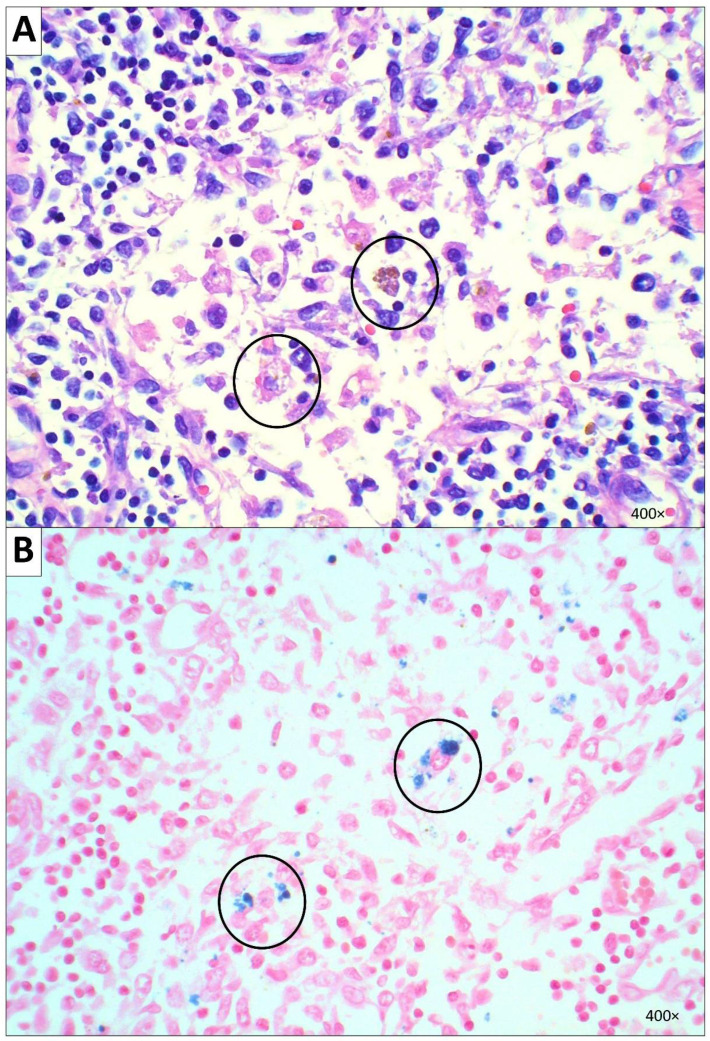
Microscopic image of subcarinal lymph nodes. (**A**) Hemophagocytosis cells with macrophages engulfing RBCs and hemosiderin are referred to with black circles (H&E, 400× magnification). (**B**) Hemophagocytosis cells were positive for Prussian blue iron stain (400× magnification).

On microscopic examination, there were intravascular AML infiltrates involving all lobes as well as diffuse intra-alveolar hemorrhages (Figure 2). Special stains for Grocott methenamine silver (GMS) and periodic acid-Schiff with diastase (PAS-D) were negative and failed to reveal evidence of fungal organisms. The subcarinal lymph nodes were enlarged (up to 2.2 cm in the greatest dimension) and showed AML infiltrates and hemophagocytosis (Figure 3).

#### 2.2.3. Gastrointestinal System

**Figure 4:**
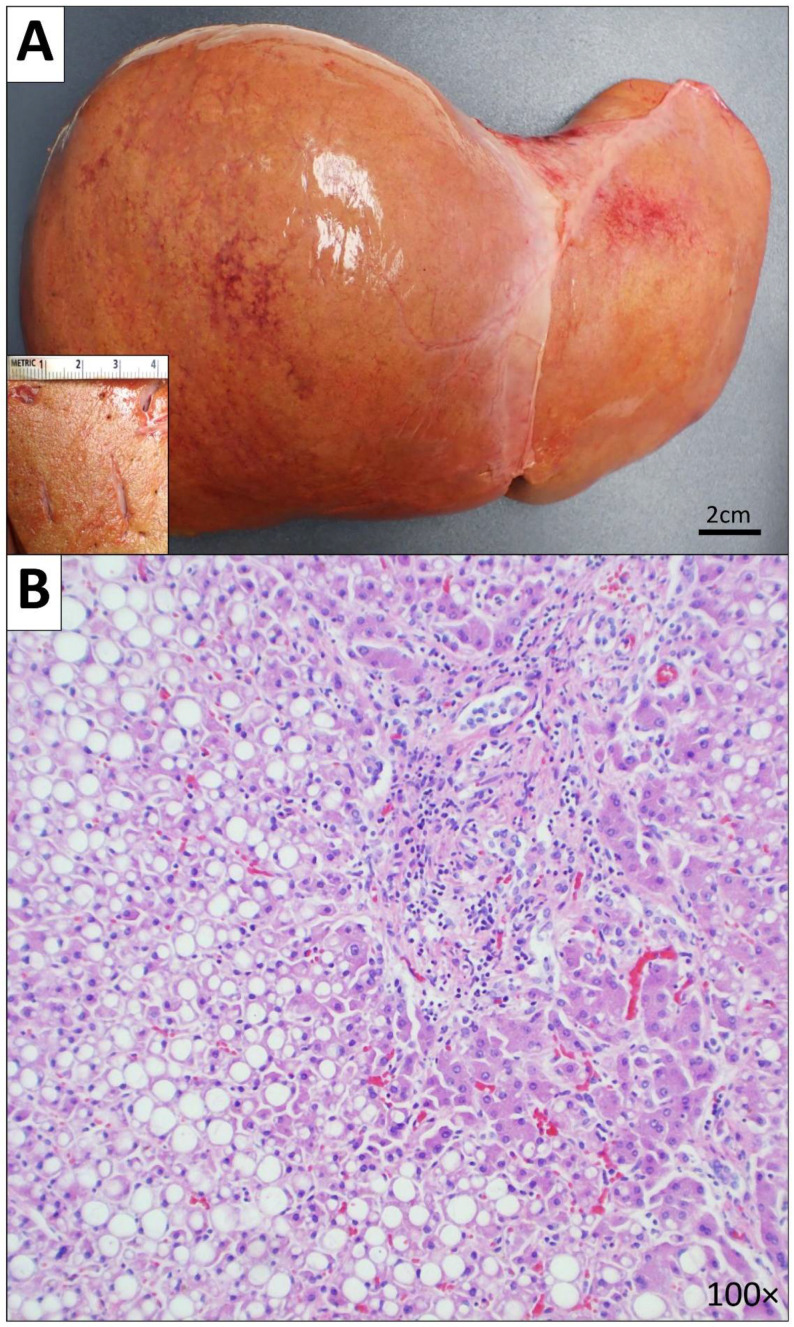
Gross and microscopic images of the liver. (**A**) Gross image of the liver. (**B**) Microscopic image of the liver showing diffuse, severe steatosis with portal, septal and focal bridging fibrosis (stage 3 fibrosis) (100× magnification).

The abdomen revealed a dry peritoneum. There was hepatomegaly (liver weighed 2700 g; normal range: 638–1782 g [7]) with diffuse, severe steatosis and portal, septal and focal bridging fibrosis (stage 3 fibrosis) (Figure 4). Supporting special stains were positive for reticulin and trichrome. Patchy, mild intrahepatocytic iron deposition (supporting special stain was positive for Prussian blue) and mild lymphocytic portal infiltrates were noted. The stomach, pancreas, and gallbladder showed autolysis. The cystic duct, hepatic duct, common bile duct, and pancreatic duct were all probe patent. The large and small bowel were grossly unremarkable, and the appendix was located in its normal anatomic position in the right lower abdomen.

#### 2.2.4. Genitourinary System

Both kidneys appeared pale with extensive autolysis and scattered cortical petechiae. Otherwise, there were no significant gross or microscopic findings. The right kidney weighed 220 g and the left kidney 240 g (normal range, right: 60–150 g, left: 62–152 g [7]). The patient had a micropenis (2.6 cm in length) and a bifid scrotum with small testicles.

#### 2.2.5. Hematopoietic System

**Figure 5:**
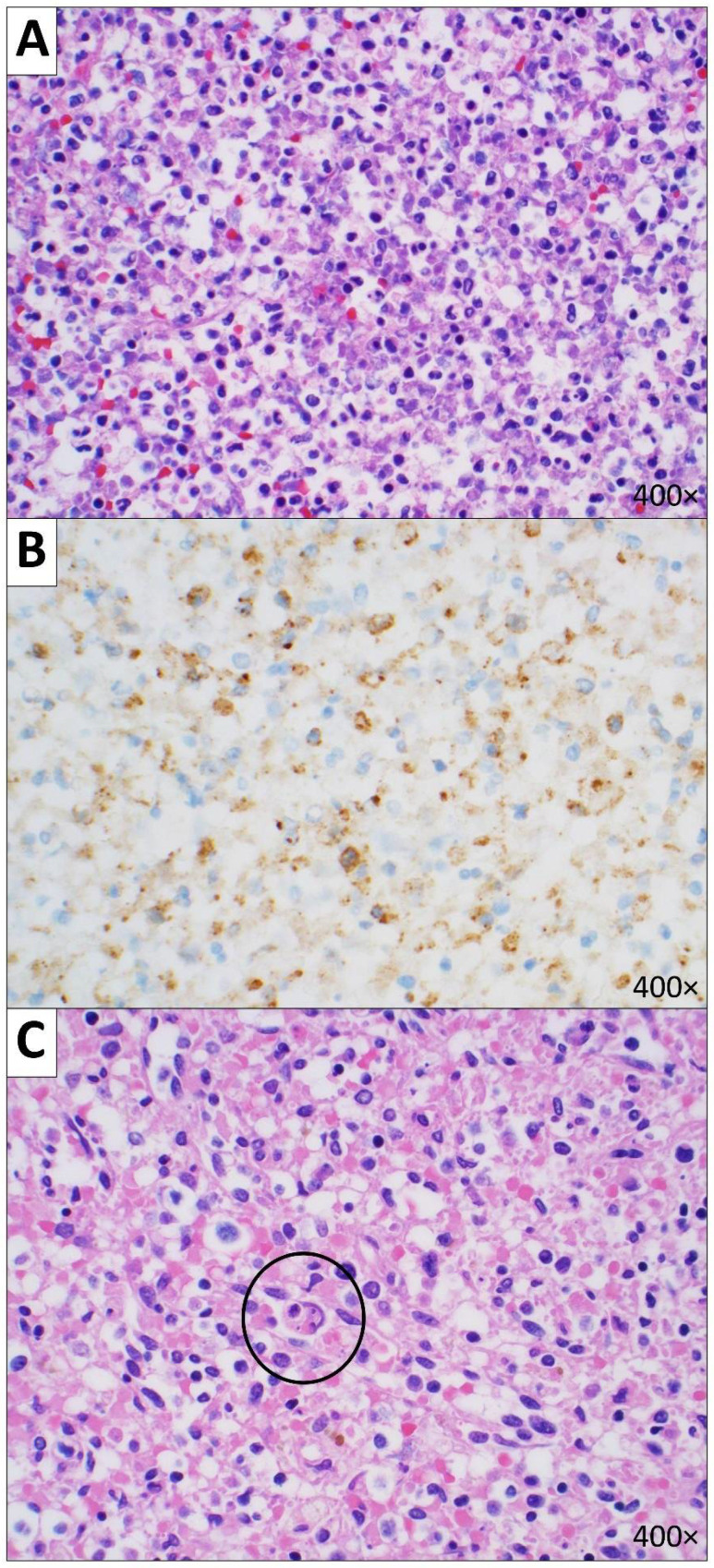
Microscopic images of the spleen. (**A**) AML infiltrates (H&E, 400× magnification). (**B**) AML cells were positive for myeloperoxidase (MPO) stain (400× magnification). (**C**) Hemophagocytosis cell with a macrophage engulfing an RBC are referred to with black circle (H&E, 400× magnification).

**Figure 6:**
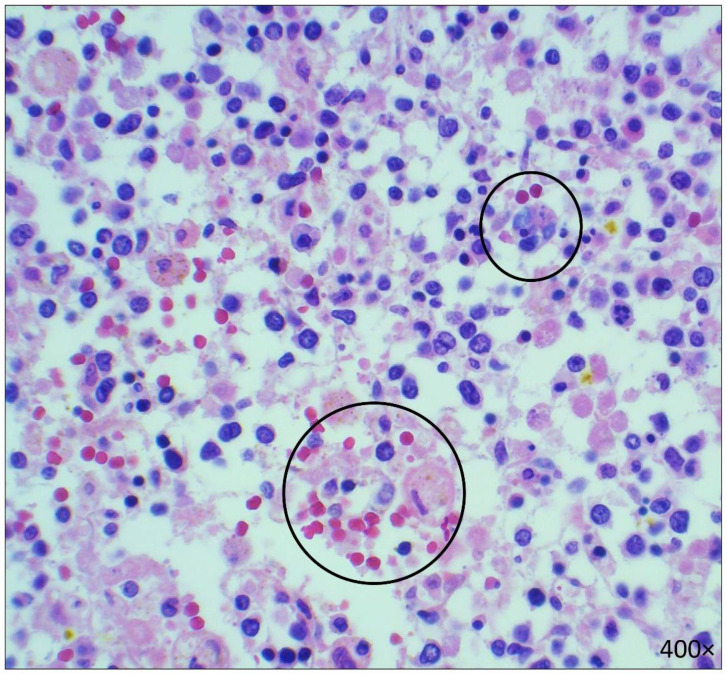
Microscopic images of the bone marrow showing hemophagocytosis, referred to with black circles (H&E, 400× magnification).

The patient had splenomegaly (490 g; reference range: 32–228 g [7]) with AML infiltrates involving the red pulp. There was also depletion of the white pulp and hemophagocytosis (supporting special stain was positive for Prussian blue) (Figure 5). The bone marrow was normocellular and showed AML infiltrates and hemophagocytosis (Figure 6).

#### 2.2.6. Endocrine System

The right and left adrenal glands were grossly and microscopically unremarkable except for autolysis. The peri-adrenal fat showed hemorrhage and focal AML infiltrates. The thyroid gland also showed intravascular AML infiltrates and cystically dilated colloid-filled follicles.

#### 2.2.7. Central Nervous System

**Figure 7:**
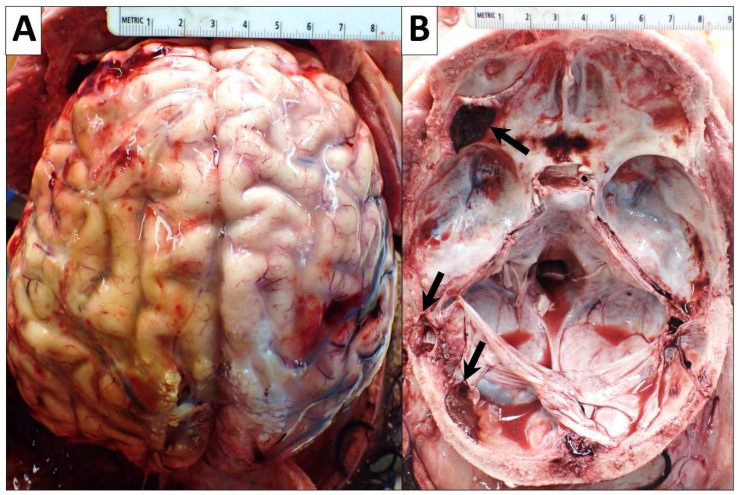
Gross images of the brain and skull. (**A**) Yellow discoloration of the convex left temporal and parietal surfaces of brain suggesting an extensive left temporal and parietal subdural hematoma (13 × 9 cm). (**B**) Base of skull (floor of the cranial cavity) showing scattered hematomas and clotted blood (black arrows).

The patient’s fresh brain weighed 1310 g (reference range for the chronological age of 12 years: 1117–1657 g [7]). There was a yellow discoloration of the convex left temporal and parietal surfaces of the brain suggesting an extensive, organized, remote left temporal and parietal subdural hematoma (13 × 9 cm). Additionally, left lateral anterior cranial fossa subdural hematoma (2 × 1.4 cm) and left parietal posterolateral epidural hematoma (2.4 × 1.3 cm) with clotted blood were seen. There were also scattered subdural hemorrhages in the sella turcica and right lateral temporal lobe (up to 1 cm) (Figure 7). The falx cerebri also had a few attached fragments of clotted blood. Microscopically, mild hypoxic–ischemic changes and vascular congestion were present. Nevertheless, no AML infiltrates were seen.

#### 2.2.8. Cultures

Postmortem lung tissue cultures were negative for fungus or bacterial organisms. No acid-fast bacilli were seen. Postmortem blood cultures were positive for enterococcus faecium and pseudomonas putida group.

## 3. Discussion

Hemophagocytic lymphohistiocytosis (HLH), also known as a hemophagocytic syndrome, is a life-threatening hyper-inflammatory systemic disorder characterized by fever, pancytopenia, splenomegaly, and the presence of hemophagocytosis in the bone marrow, liver, and lymph nodes [1]. It was first described in 1939 by Scott and Robb-Smith, who referred to it as histiocytic medullary reticulosis [8]. HLH is due to immunologic dysregulation where there is abnormal or impaired function of the natural killer (NK) cells and cytotoxic T lymphocytes (CTL), leading to an excessive and uncontrolled immune response [9,10]. The diagnosis of HLH is based on standard criteria proposed by Henter et al. in 2004 [1]. HLH can be either primary (hereditary) or secondary [2,3]. The former is associated with a wide array of genetically heterogeneous disorders and is typically triggered by common infections [4]. The latter typically occurs after some form of immunosuppression such as occurs with malignancy [5]. Malignancy-associated HLH is typically associated with lymphoid neoplasms and leukemias, although solid tumors may serve as the inciting malignancy as well [2,3]. Our case is that of secondary HLH occurring in the setting of t-AML. The patient presented with febrile neutropenia and was found to have hepatosplenomegaly on physical examination. During hospitalization, the patient developed multiple infections and septic shock. Laboratory tests supported the diagnosis of HLH. The patient had hemorrhagic pericardial effusion, deteriorated, and eventually expired. Autopsy findings revealed AML cell infiltrates and hemophagocytosis in the heart, pericardial effusion, lungs, lymph nodes, spleen, and bone marrow tissues.

Acute lymphoblastic leukemia (ALL) is the most common pediatric cancer, representing over one-third of all pediatric cancer diagnoses [11]. The annual incidence of pediatric ALL in the United States is approximately 3.4 cases per 100,000 children, with the peak incidence occurring in children aged 2-to-5 years and tapering off with increasing age [11,12]. A similar incidence appears worldwide [13]. ALL is a malignancy of bone marrow lymphocytes characterized by the uncontrolled proliferation of abnormal immature cells, which ultimately replace the normal bone marrow with dysfunctional cells and their progenitors [14,15]. This results in lymphocytopenia, recurrent infections, pallor, easy bruising and bleeding, bone pain, muscle pain, hepatosplenomegaly, and/or lymphadenopathy [11].

The prognosis of ALL remains largely linked to the patient’s age. Approximately 90% of patients aged 1-to-10 years achieve long-term survival, with life expectancy decreasing to 40–50% in adolescents and young adults [16]. The exact age at which the prognosis markedly worsens is not clear. ALL affects white children more than black children and shows a slight male predominance [17,18]. The cause of ALL remains largely unknown [19,20,21,22,23]. There have been no established links between environmental exposure, ionizing radiation, and viral exposure. While some cases of the disease have been associated with genetic disorders and congenital immunodeficiencies, this link remains insignificant [23].

In contrast to the lymphocytic hyper-proliferation seen in ALL, acute myeloid leukemia (AML) is a neoplasm resulting in the malignant transformation of hematopoietic stem cells and progenitor cells, which eventually leads to an uncontrolled proliferation of abnormal cells [24]. The symptoms of AML are very similar to ALL and include easy bruising and bleeding, fever, malaise, muscle pain, bone pain, hepatosplenomegaly, and lymphadenopathy [25]. A high degree of suspicion must be exercised in order to make the diagnosis of HLH [25]. One key difference between the development of ALL and AML is that several known factors are associated with the development of AML including radiation, exposure to environmental toxins and drugs, as well as genetic mutations and syndromes [26,27]. While there appears to be an association with these variables, the exact connection is still unknown since the majority of people with these factors do not develop AML [26,27].

As with ALL, little is known regarding the exact etiology of AML in most patients. However, there are several types of AML in which a clear antecedent is identified, and these are considered genetically distinct from de novo AML [28]. Therapy-related AML (t-AML) can occur following treatment with cytotoxic chemotherapy, radiation therapy, anthracyclines, and topoisomerase-II inhibitors [28,29]. Topoisomerase-II inhibitors alone account for approximately 6.8% of all AML cases [29]. In our case, there is documentation that the patient received anthracycline chemotherapy and eventually developed t-AML. T-AML has a delayed presentation after exposure to the inciting therapy, which ranges from 1 to 7 years post-exposure, depending on the treatment modality or chemotherapeutic agent used [27,29,30,31]. The presence of t-AML portends an independently increased risk of death as compared to de novo AML [29]. Additionally, in contrast to de novo AML, patient age seems to have a direct impact on the prognosis of t-AML, with younger patients achieving worse clinical outcomes that include death [29].

Nearly all patients with HLH eventually develop multi-organ involvement, primarily of the liver, CNS, cardiovascular system, respiratory system, kidneys, and skin [2,32]. Hepatitis is extremely common and presents as elevated liver enzymes (AST, ALT, GGT), lactate dehydrogenase (LDH), and bilirubin [2,32]. Hepatic dysfunction and disseminated intravascular coagulopathy can also result in elevated triglycerides and impaired coagulation [2,32,33]. Common histopathological findings of the liver among patients with HLH include sinusoidal dilatation, hepatocellular necrosis, endothelialitis, and steatosis [34,35]. In our case, the liver was enlarged and showed diffuse, severe steatosis with portal, septal and focal bridging fibrosis.

Neurologic findings are very common and can present as altered mental status, seizures, or ataxia [2,36]. Bleeding is common due to a combination of hepatic dysfunction, thrombocytopenia from bone marrow dysfunction, or abnormal platelets [2,33], which explains the extensive, scattered hemorrhages and subdural and epidural hematomas we found in our patient. Renal dysfunction may present as hyponatremia and can progress to renal failure requiring dialysis [37]. Severe cardiac dysfunction can occur due to either hyper-inflammation or drug toxicity [2]. In our case, a critical complication that the patient experienced was repetitive hemorrhagic pericardial effusions, which recurred despite continuous drainage. On autopsy, the pericardial sac had extensive and diffuse hemorrhagic fibrinous and fibrous adhesions, hemorrhage, and clotted blood, as well as fibrinous exudates, which showed the presence of hemophagocytosis. There were also AML infiltrates associated with hemorrhage and necrosis that extensively involved the pericardial fibroadipose tissue, epicardium, and myocardium. Skin manifestations in HLH vary and can present as a rash, edema, erythema, petechiae, or purpura [37]. Our patient had no significant skin findings. Respiratory involvement manifests as an urgent need for ventilation and indicates acute respiratory distress-like syndrome from worsening HLH or infection [37]. Our patient developed a fungal infection of the lungs during the course of hospitalization. He was intubated and put on V-ECMO. Autopsy findings demonstrated severe bilateral pulmonary congestion and edema. Importantly, intravascular AML infiltrates were found involving all lung lobes bilaterally.

While infection and inflammation tend to be noticed and treated, HLH remains highly undiagnosed due to the lack of available testing for immune system dysregulation and impaired activation that occurs in this rare disorder [2]. Consequently, the mortality rate is as high as 50% for HLH patients when only supportive care is provided [2]. These patients often die from overwhelming infection, uncontrolled hyper-inflammation, and multi-organ failure. Being cognizant of and maintaining a high index of suspicion in patients with known risk factors coupled with prompt diagnosis and immediate implementation of aggressive supportive therapy is imperative in the management of this condition. HLH is often accompanied by a viral infection, most commonly Epstein-Barr virus (EBV), cytomegalovirus (CMV), herpes simplex virus and other herpes viruses, parvovirus, measles virus, influenza virus, and HIV [38]. Less frequently, HLH can be seen in conjunction with a concomitant bacterial, parasitic, or fungal infection [4,38]. In patients with a history of anti-TNF therapy who subsequently develop HLH, surveillance for bacterial infections is strongly recommended [39].

Similar to other types of HLH, malignancy-associated HLH is often accompanied by an acute infectious trigger [3]. One study detected—using PCR—the presence of BK virus in 54%, HHV-6 in 33%, EBV in 28%, CMV in 24%, adenovirus in 17%, and parvovirus-type B in 17% in malignancy-associated HLH patients with a hematologic malignancy [3]. When associated with a malignancy, HLH is more immediately life-threatening than the malignancy itself [40,41,42]. The treatment of HLH consists of corticosteroids and/or intravenous immunoglobulins along with targeted antimicrobial therapy. However, the overall prognosis remains poor in all affected patients regardless of age [40,41,42], with a mortality rate exceeding 50% as shown in one study [43]. There appears to be a strong association between malignancy-associated HLH and adult AML and ALL, with detection of HLH in up to 18% and 4% of these cases, respectively [3]. However, the incidence of malignancy-associated HLH in pediatric AML and ALL cases is currently unknown and remains a topic of interest.

## 4. Conclusions

In conclusion, early diagnosis of HLH is essential and should be considered in patients with AML who present with pancytopenia. While infection and inflammation tend to be noticed and treated, HLH remains frequently underdiagnosed due to the lack of testing for immune system dysregulation and impaired activation because of the rarity of the disorder. Diagnostic vigilance and prompt implementation of treatment are vital for the management of HLH.

## Figures and Tables

**Table 1:** Diagnostic criteria of hemophagocytic lymphohistiocytosis. Adapted from the standard diagnostic criteria proposed in 2004 by Henter et al. HLH-2004 [1].

Diagnosis Is Established If One of Either (I) or (II) Is Fulfilled
(I) Molecular diagnosis consistent with HLH
(II) Clinicopathologic criteria for HLH fulfilled (5 out of the 8 criteria shown below)
1. Fever ≥ 38.5 °C for ≥ 7 days
2. Splenomegaly ≥ 3 finger breadth below the left subcostal margin
3. Cytopenias affecting ≥ 2 of 3 lineages in peripheral blood
• Hemoglobin < 9 g/L
• Platelets < 100 × 10^9^/L
• Absolute neutrophil count < 1.0 × 10^9^/L
4. Hypertriglyceridemia and/or hypofibrinogenemia
• Fasting triglycerides ≥ 265 mg/dL, Fibrinogen ≥ 1.5 g/L
5. Hemophagocytosis in the bone marrow or spleen or lymph node
6. Low or absent NK cell activity (according to the local laboratory reference)
7. Ferritin ≥ 500 μg/L
8. Soluble CD25 (sIL-2 receptor) ≥ 2400 U/mL

## Data Availability

Not applicable.

## References

[1] Henter J.-I., Horne A., Aricó M., Egeler R.M., Filipovich A.H., Imashuku S., Ladisch S., McClain K., Webb D., Winiarski J. (2007). HLH-2004: Diagnostic and therapeutic guidelines for hemophagocytic lymphohistiocytosis. Pediatr. Blood Cancer.

[2] Jordan M.B., Allen C.E., Weitzman S., Filipovich A.H., McClain K.L. (2011). How I treat hemophagocytic lymphohistiocytosis. Blood.

[3] Strenger V., Merth G., Lackner H., Aberle S.W., Kessler H.H., Seidel M.G., Schwinger W., Sperl D., Sovinz P., Karastaneva A. (2018). Malignancy and chemotherapy induced haemophagocytic lymphohistiocytosis in children and adolescents—A single centre experience of 20 years. Ann. Hematol..

[4] Gosh J.B., Roy M., Bala A. (2009). Infection Associated with Hemophagocytic Lymphohisticytosis triggered by nosocomial Infection. Oman Med. J..

[5] Karlsson T. (2015). Secondary haemophagocytic lymphohistiocytosis: Experience from the Uppsala University Hospital. Upsala J. Med. Sci..

[6] Wang H., Xiong L., Tang W., Zhou Y., Li F. (2017). A systematic review of malignancy-associated hemophagocytic lymphohistiocytosis that needs more attentions. Oncotarget.

[7] Molina D.K., Pinneri K., Stash J.A., Li L., Vance K., Cross C. (2019). Organ Weight Reference Ranges for Ages 0 to 12 Years. Am. J. Forensic Med. Pathol..

[8] Scott R.B., Robb-Smith A. (1939). Histiocytic Medullary Reticulosis. Lancet.

[9] Egeler R.M., Shapiro R., Loechelt B., Filipovich A. (1996). Characteristic Immune Abnormalities in Hemophagocytic Lymphohistiocytosis. J. Pediatr. Hematol. Oncol..

[10] Vrotsos E., Soaita M., Khatib Z., Brathwaite C., Filipovich A., Robinson M.J., Castellano-Sanchez A.A. (2013). Atypical clinical presentation of primary hemophagocytic lymphohistiocytosis with a novel perforin1 gene mutation. J. Hematop..

[11] Clarke R.T., Van den Bruel A., Bankhead C., Mitchell C.D., Phillips B., Thompson M.J. (2016). Clinical presentation of childhood leukaemia: A systematic review and meta-analysis. Arch. Dis. Child..

[12] Ward E., DeSantis C., Robbins A., Kohler B., Jemal A. (2014). Childhood and adolescent cancer statistics, 2014. CA Cancer J. Clin..

[13] Steliarova-Foucher E., Colombet M., Ries L.A.G., Moreno F., Dolya A., Bray F., Hesseling P., Shin H.Y., Stiller C.A. (2017). International incidence of childhood cancer, 2001–2010: A population-based registry study. Lancet Oncol..

[14] Roberts K.G. (2018). Genetics and prognosis of ALL in children vs adults. Hematol. Am. Soc. Hematol. Educ. Program..

[15] Onciu M. (2009). Acute Lymphoblastic Leukemia. Hematol. Clin. N. Am..

[16] Ribera J.-M., Oriol A. (2009). Acute Lymphoblastic Leukemia in Adolescents and Young Adults. Hematol. Oncol. Clin. N. Am..

[17] Kroll M.E., Stiller C.A., Murphy M.F.G., Carpenter L.M. (2011). Childhood leukaemia and socioeconomic status in England and Wales 1976–2005: Evidence of higher incidence in relatively affluent communities persists over time. Br. J. Cancer.

[18] Feng Q., de Smith A.J., Vergara-Lluri M., Muskens I.S., McKean-Cowdin R., Kogan S., Brynes R., Wiemels J.L. (2021). Trends in Acute Lymphoblastic Leukemia Incidence in the United States by Race/Ethnicity From 2000 to 2016. Am. J. Epidemiol..

[19] Greaves M. (2006). Infection, immune responses and the aetiology of childhood leukaemia. Nat. Rev. Cancer.

[20] Krestinina L., Davis F.G., Schonfeld S., Preston D.L., Degteva M., Epifanova S., Akleyev A.V. (2013). Leukaemia incidence in the Techa River Cohort: 1953–2007. Br. J. Cancer.

[21] Urayama K.Y., Ma X., Selvin S., Metayer C., Chokkalingam A.P., Wiemels J.L., Does M., Chang J., Wong A., Trachtenberg E. (2011). Early life exposure to infections and risk of childhood acute lymphoblastic leukemia. Int. J. Cancer.

[22] Boothe V.L., Boehmer T.K., Wendel A.M., Yip F.Y. (2014). Residential traffic exposure and childhood leukemia: A systematic review and meta-analysis. Am. J. Prev. Med..

[23] Buitenkamp T.D., Izraeli S., Zimmermann M., Forestier E., Heerema N.A., van den Heuvel-Eibrink M.M., Pieters R., Korbijn C.M., Silverman L.B., Schmiegelow K. (2014). Acute lymphoblastic leukemia in children with Down syndrome: A retrospective analysis from the Ponte di Legno study group. Blood.

[24] Chen J., Glasser C. (2020). New and Emerging Targeted Therapies for Pediatric Acute Myeloid Leukemia (AML). Children.

[25] Meyers C.A., Albitar M., Estey E. (2005). Cognitive impairment, fatigue, and cytokine levels in patients with acute myelogenous leukemia or myelodysplastic syndrome. Cancer.

[26] Bhatia S., Neglia J. (1995). Epidemiology of Childhood Acute Myelogenous Leukemia. J. Pediatr. Hematol. Oncol..

[27] Puumala S.E., Ross J.A., Aplenc R., Spector L.G. (2013). Epidemiology of childhood acute myeloid leukemia. Pediatr. Blood Cancer.

[28] Granfeldt Østgård L.S., Medeiros B.C., Sengeløv H., Nørgaard M., Andersen M.K., Dufva I.H., Friis L.S., Kjeldsen E., Marcher C.W., Preiss B. (2015). Epidemiology and Clinical Significance of Secondary and Therapy-Related Acute Myeloid Leukemia: A National Population-Based Cohort Study. J. Clin. Oncol..

[29] Sardou-Cezar I., Lopes B.A., Andrade F.G., Fonseca T.C.C., Fernandez T.D.S., Larghero P., de Souza R.Q., Loth G., Ribeiro L.L., Bonfim C. (2021). Therapy-related acute myeloid leukemia with KMT2A-SNX9 gene fusion associated with a hyperdiploid karyotype after hemophagocytic lymphohistiocytosis. Cancer Genet..

[30] Tebbi C.K., London W.B., Friedman D., Villaluna D., De Alarcon P.A., Constine L.S., Mendenhall N.P., Sposto R., Chauvenet A., Schwartz C.L. (2007). Dexrazoxane-Associated Risk for Acute Myeloid Leukemia/Myelodysplastic Syndrome and Other Secondary Malignancies in Pediatric Hodgkin’s Disease. J. Clin. Oncol..

[31] Pedersen-Bjergaard J., Philip P. (1991). Two different classes of therapy-related and de-novo acute myeloid leukemia?. Cancer Genet. Cytogenet..

[32] Palazzi D.L., McClain K., Kaplan S.L. (2003). Hemophagocytic Syndrome in Children: An Important Diagnostic Consideration in Fever of Unknown Origin. Clin. Infect. Dis..

[33] Fukaya S., Yasuda S., Hashimoto T., Oku K., Kataoka H., Horita T., Atsumi T., Koike T. (2008). Clinical features of haemophagocytic syndrome in patients with systemic autoimmune diseases: Analysis of 30 cases. Rheumatology.

[34] Favara B.E. (1996). Histopathology of the Liver in Histiocytosis Syndromes. Pediatr. Pathol. Lab. Med..

[35] Kerguenec C., Hillaire S., Molinie V., Gardin C., Degott C., Erlinger S., Valla D. (2001). Hepatic manifestations of hemophagocytic syndrome: A study of 30 cases. Am. J. Gastroenterol..

[36] Jovanovic A., Kuzmanovic M., Kravljanac R., Micic D., Jovic M., Gazikalovic S., Pasic S. (2014). Central Nervous System Involvement in Hemophagocytic Lymphohistiocytosis: A Single-Center Experience. Pediatr. Neurol..

[37] Ramos-Casals M., Brito-Zerón P., López-Guillermo A., Khamashta M.A., Bosch X. (2014). Adult haemophagocytic syndrome. Lancet.

[38] Mostaza-Fernández J.L., Guerra Laso J., Carriedo Ule D., Ruiz de Morales J.M. (2014). Hemophagocytic lymphohistiocytosis associated with viral infections: Diagnostic challenges and therapeutic dilemmas. Rev. Clin. Esp..

[39] Brito-Zerón P., Bosch X., Pérez-De-Lis M., Pérez-Álvarez R., Fraile G., Gheitasi H., Retamozo S., Bové A., Monclús E., Escoda O. (2016). Infection is the major trigger of hemophagocytic syndrome in adult patients treated with biological therapies. Semin. Arthritis Rheum..

[40] Parikh S.A., Kapoor P., Letendre L., Kumar S., Wolanskyj A.P. (2014). Prognostic Factors and Outcomes of Adults With Hemophagocytic Lymphohistiocytosis. Mayo Clin. Proc..

[41] Otrock Z.K., Eby C.S. (2015). Clinical characteristics, prognostic factors, and outcomes of adult patients with hemophagocytic lymphohistiocytosis. Am. J. Hematol..

[42] Schram A.M., Comstock P., Campo M., Gorovets D., Mullally A., Bodio K., Arnason J., Berliner N. (2016). Haemophagocytic lymphohistiocytosis in adults: A multicentre case series over 7 years. Br. J. Haematol..

[43] Brito-Zerón P., Kostov B., Moral-Moral P., Martínez-Zapico A., Díaz-Pedroche C., Fraile G., Pérez-Guerrero P., Fonseca E., Robles A., Vaquero-Herrero M.P. (2018). Prognostic Factors of Death in 151 Adults With Hemophagocytic Syndrome: Etiopathogenically Driven Analysis. Mayo Clin. Proc. Innov. Qual. Outcomes.

